# Spontaneous gas gangrene of the pancreas: extremely rare condition

**DOI:** 10.1093/jscr/rjae133

**Published:** 2024-04-04

**Authors:** Bacem Zaidi, Sihem Sindi, Leila Belgacem, Zied Mansi, Wael Gazzah, Khalil Ben Salah

**Affiliations:** University of Sousse, Sousse 4200, Tunisia; Les Aghlabides Hospital, Kairouan 3100, Tunisia; University of Sousse, Sousse 4200, Tunisia; Les Aghlabides Hospital, Kairouan 3100, Tunisia; University of Sousse, Sousse 4200, Tunisia; Les Aghlabides Hospital, Kairouan 3100, Tunisia; University of Sousse, Sousse 4200, Tunisia; Les Aghlabides Hospital, Kairouan 3100, Tunisia; Tunisian Society of Orthopedic and Traumatology Surgery, Tunis 1000, Tunisia; University of Sousse, Sousse 4200, Tunisia; Les Aghlabides Hospital, Kairouan 3100, Tunisia; Urology Department, Kairouan 3100, Tunisia; University of Sousse, Sousse 4200, Tunisia; Les Aghlabides Hospital, Kairouan 3100, Tunisia

**Keywords:** necrotizing pancreatitis, *Clostridium perfringens*, pancreatic gas gangrene, spontaneous pneumoperitoneum

## Abstract

Acute pancreatitis is a common condition, only occasionally leading to necrosis of the pancreas. In instances where abscess formation takes place, the predominant microbial profile involves both aerobic and anaerobic enteric species. We present the case of a patient with clostridial emphysematous pancreatitis who developed pneumoperitoneum without associated visceral perforation.

## Introduction

Necrotizing pancreatitis caused by *Clostridium perfringens* is a rare and serious condition associated with high morbidity and mortality. Unusual presentation on CT scan with pneumoperitoneum is infrequent and often linked to a poor prognosis [1]. Typically, surgical intervention for acute pancreatitis is recommended in the presence of infected necrosis, acute abdomen, or abdominal compartment syndrome. Diagnosis confirmation can involve CT-guided percutaneous aspiration or intraoperative methods [2]. In this case, successful management of *C. perfringens*-induced acute pancreatitis involved resuscitation and surgical debridement of necrotic pancreatic tissue [3].

## Case report

**Figure 1:**
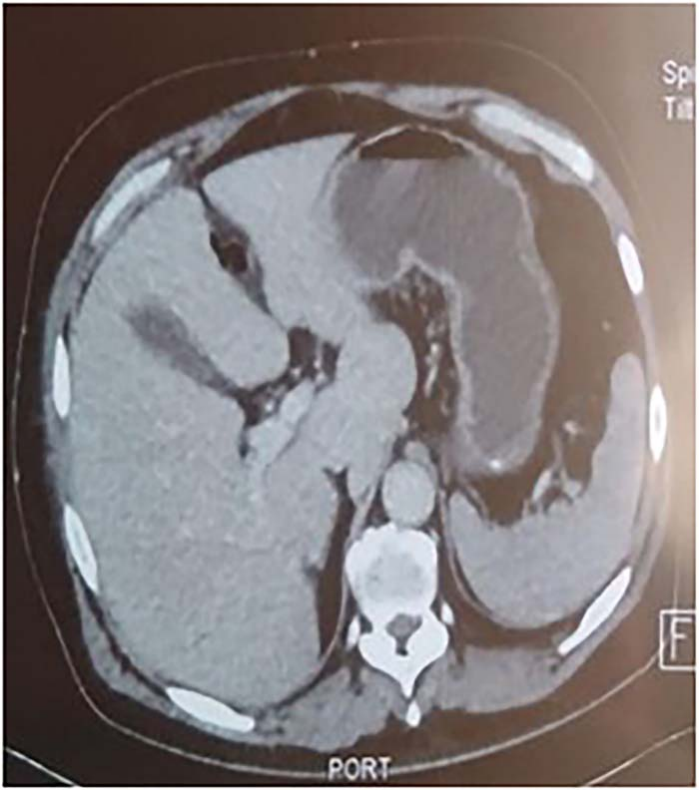
Pneumoperitoneum.

**Figure 2:**
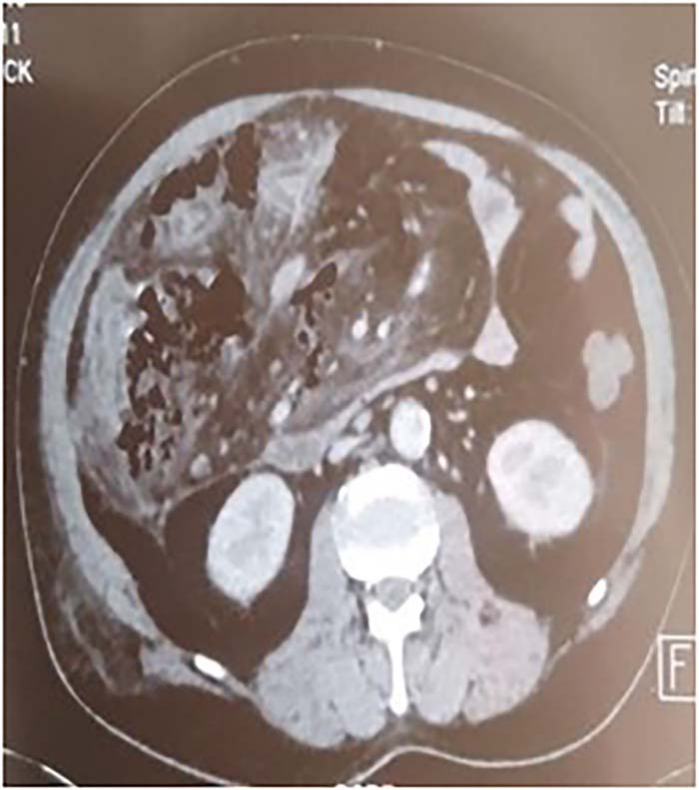
Pancreatic necrosis.

**Figure 3:**
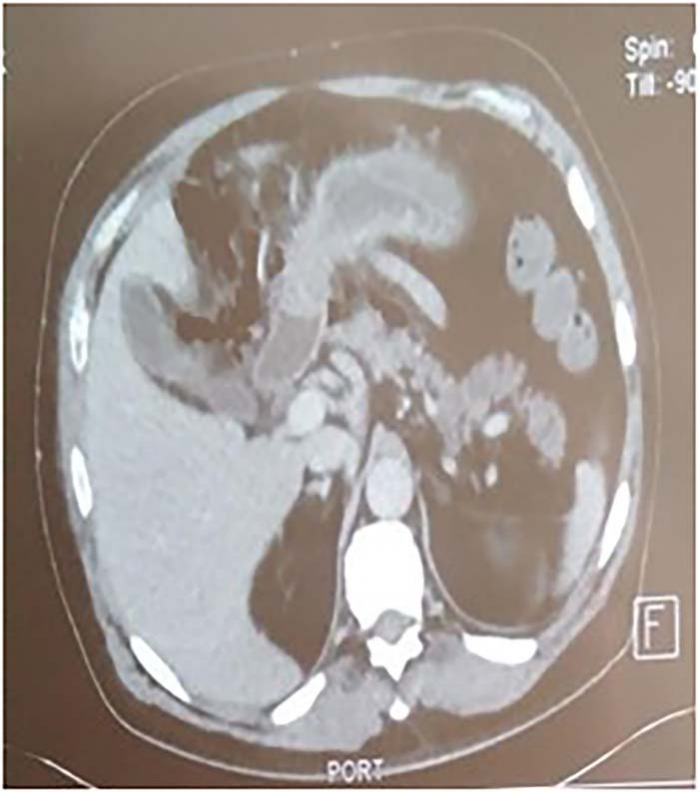
Thickening of the mesentery root.

A 53-year-old man with no known medical history was admitted to the hospital because of epigastric pain persisting for 24 h, accompanied by weakness. Physical examination revealed a temperature of 39°C, severe dehydration, poor peripheral perfusion, a pulse of 110 bpm, blood pressure at 10/6 mm/Hg, a painful and tympanic abdomen without peristalsis, with tenderness over the epigastric region. Laboratory tests showed a lipase level of 667 U/l, sodium at 140 mmol/l, potassium at 4.5 mmol/l, creatinine at 106 μmol/l, urea at 6.5 mmol/l, alanine aminotransferase at 161 U/l, aspartate aminotransferase at 85 U/l, an elevated leukocyte count at 29,3 × 10^9^/l. Abdominal CT with contrast revealed pancreatic necrosis with gas surrounding the pancreas, as well as pneumoperitoneum and in the right anterior pararenal space. Thickening of the mesentery root with lymph node enlargement (Figs 1–3).

**Figure 4:**
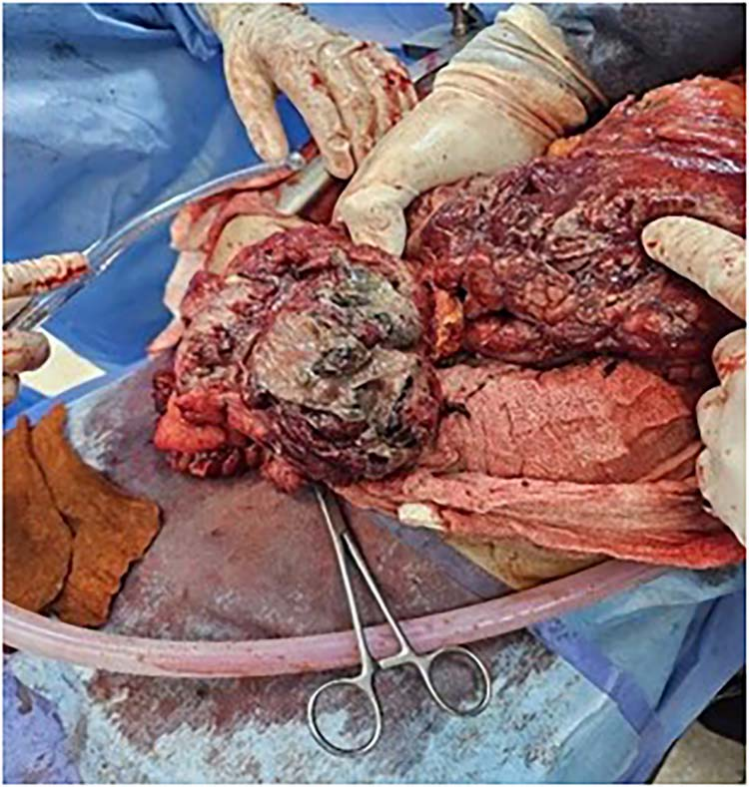
Omentum gangrene.

**Figure 5:**
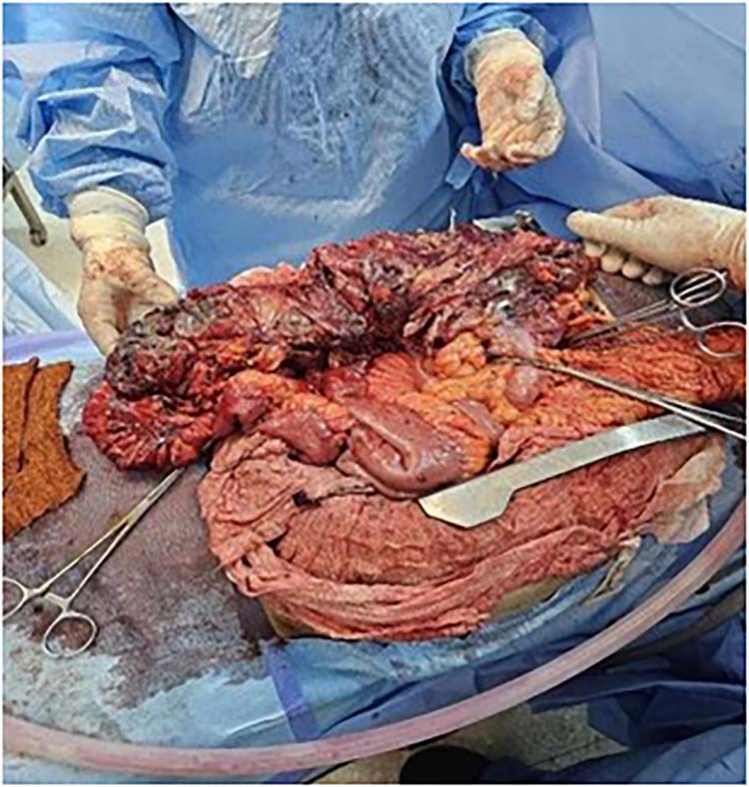
Right colon gangrene.

The patient was treated with fluids, imipenem, and underwent emergency surgery. Laparotomy showed purulent fluid in the peritoneal cavity with diffuse gangrene involving the distal small bowel and the right colon. The mesentery root was markedly thickened and infiltrated, site of multiple purulent collections. Gangrene was present in the omental bursa with destruction of the cephalic part of the pancreas. The procedure included necrosectomy extensive cleansing, right colectomy with double ileostomy and colostomy, and drainage (Figs 4 and 5). Unfortunately, the patient succumbed to septic shock a few hours after the intervention. The purulent fluid sample returned after examination and culture positive to *C. perfringens*.

## Discussion

Gas gangrene of the pancreas secondary to *C. perfringens* infection is an extremely rare and severe form of acute pancreatitis [1, 4]. It can occur spontaneously without prior instrumental manipulations or known underlying diseases, characterized by the presence of spontaneous pneumoperitoneum without visceral perforation [5, 6].

Infections during necrotizing hemorrhagic pancreatitis occur in only 33% of cases and are associated with a mortality rate ranging from 14% to 62% [3]. Commonly implicated microorganisms include *Escherichia coli*, Enterococcus, Streptococcus, Klebsiella, and Enterobacter, with not uncommon polymicrobial infections. Although anaerobic infections are less frequent, they are often associated with significant morbidity and mortality, attributed to the aggressive microbial agent *C. perfringens* [2, 3].

The source and route of infection are not fully understood and may be hematogenous, lymphatic, or result from transmural penetration from the colon, retrograde duodenal infection, or from infected bile [1, 3].

*Clostridium perfringens*, the organism responsible for classic myonecrosis gas gangrene, is a gram-positive, spore-forming, strictly anaerobic bacillus that is part of the normal human intestinal flora. It produces the cytopathic α toxin, which directly degrades cell membranes, leading to massive hemolysis and tissue necrosis, resulting in gas accumulation in the pancreatic bed observed on CT, aiding in the early diagnosis of clostridial gas gangrene of the pancreas, as seen in our case [2, 5].

The diagnosis of *C. perfringens* pancreatitis is challenging because of its high morbidity and rapid progression to death [7]. The decision to undergo surgical intervention remains problematic, with medical management generally recommended initially unless the necrotic pancreas is infected [1].

In our case, CT plays a significant role in therapeutic management, and the presence of extra-digestive gas motivates the surgical indication [1, 3, 6].

Neither the presence nor the extent of pancreatic necrosis is an absolute indication for debridement, though morbidity and mortality increase with the extent of necrosis. The therapeutic strategy involves emergency laparotomy: necrosectomy, excision of necrotic organs, extensive drainage, and the use of intravenous carbapenem antibiotics, including imipenem, panipenem, and meropenem. The key to effectively treating this serious infectious disease lies in early diagnosis, open drainage, and the appropriate use of carbapenems. The use of hyperbaric oxygen was considered but rejected, as the current clostridial infection was deep-seated [3, 8, 9].

## Conclusion

Acute pancreatitis is a serious disease and can become fatal if associated with overinfection with *C. perfringens*. In this case, no therapeutic strategy is codified. But it is mainly based on antibiotic therapy and in some cases the recourse to surgery is inevitable but at the cost of heavy mortality.
